# Relationships between menstrual status and obesity phenotypes in women: a cross-sectional study in northern China

**DOI:** 10.1186/s12902-020-00577-6

**Published:** 2020-06-22

**Authors:** Xueyu Chen, Hui Xi, Long Ji, Weihua Liu, Fengxue Shi, Yanru Chen, Xiaohui Wang, Wenran Zhang, Xinxia Sui, Xiaojun Wang, Haitao Zhang, Huamin Liu, Dong Li

**Affiliations:** 1 School of public health, Shandong First Medical University & Shandong Academy of Medical Sciences, Tai’an, Shandong Province China; 2 Department of Cardiology, Peking University International Hospital, Beijing, China; 3 School of nursing, Shandong First Medical University & Shandong Academy of Medical Sciences, Tai’an, Shandong Province China; 4 The Second Affiliated Hospital of Shandong First Medical University, Tai’an, Shandong Province China; 5 Taian Maternal and Child Health Hospital, Tai’an, Shandong Province China; 6 School of Public Health, Southern Medical University, Guangzhou, Guangdong Province China

**Keywords:** Menstruation, Obesity, Phenotypes, Postmenopausal

## Abstract

**Background:**

One of most important concerns of postmenopausal women is obesity. The relationships between menstruation status and obesity phenotypes are unclear. This study aimed to assess the associations between menstrual status and different obesity phenotypes in women.

**Methods:**

In total, 5373 women aged ≥40 years were recruited from the Jidong and Kailuan communities. Basic information was collected via clinical examination, laboratory testing and standardized questionnaires. The women were stratified into the following three groups: menstrual period, menopausal transition period and postmenopausal period. General obesity was defined as a body mass index (BMI) of ≥28 kg/m^2^. Central obesity was defined as a waist-to-hip ratio (WHR) of > 0.85. Visceral obesity was defined as the presence of nonalcoholic fatty liver disease (NAFLD) and increased pericardial fat volume (PFV).

**Results:**

The numbers of women in the menstrual, menopausal transition, and postmenopausal periods were 2807 (52.2%), 675 (12.6%) and 1891 (35.2%), respectively. The adjusted odds ratio (OR) and 95% confidence interval (CI) for central obesity among women in the menopausal transition and postmenopausal periods compared with women in the menstrual period were 0.99 (0.82–1.19) and 1.52 (1.26–1.84), respectively. The OR for NAFLD among postmenopausal women was 1.78 (1.44–2.20). The adjusted β-coefficient (standard error, SE) for PFV among postmenopausal women was 41.25 (7.49). The adjusted OR for general obesity among postmenopausal women was 1.01 (0.77–1.34).

**Conclusions:**

This study demonstrated that menopause is an independent risk factor for central and visceral obesity but not general obesity.

## Background

Obesity is a common risk factor for many diseases, such as metabolic syndrome, coronary heart disease, and type 2 diabetes [1, 2]. The global incidence of obesity is 13%, and the number of obese people has increased rapidly in China, gravely impacting the health of the Chinese population [3, 4]. Obesity is caused by excessive accumulation of fat and can be classified according to the location of fat accumulation as general obesity, central obesity or visceral obesity. General obesity reflects the overall body mass, i.e., a large lean (muscle) mass. Central obesity has been shown to be an accurate measure of abdominal fat accumulation and a better predictor of morbidity and mortality than general obesity [5]. Visceral obesity is a well-known risk factor for many diseases, such as metabolic disease, hemodynamic disease, type 2 diabetes, and stroke [6, 7].

Menstrual changes are natural physiological processes in women and include the menstrual period, the menopausal transition period and the postmenopausal period. Women in different stages of menstruation undergo different physical and psychological changes [8]. Compared to women in the other two periods, women in the postmenopausal period tend to have a higher risk of disease, probably due to a sharp decrease in the levels of sex hormones after menopause [9]. Increased oestrogen levels have been reported to affect weight gain by increasing energy expenditure and by regulating metabolism and the distribution of adipose tissue [10].

Several studies have reported associations between obesity and menstrual status [11, 12]. To our knowledge, no previous study has evaluated the association of menstrual status with different obesity phenotypes. This study aimed to explore the associations between menstrual status and the locations of fat deposits in Chinese women, thereby improving and promoting women’s health.

## Methods

### Study design and population

**Fig. 1:**
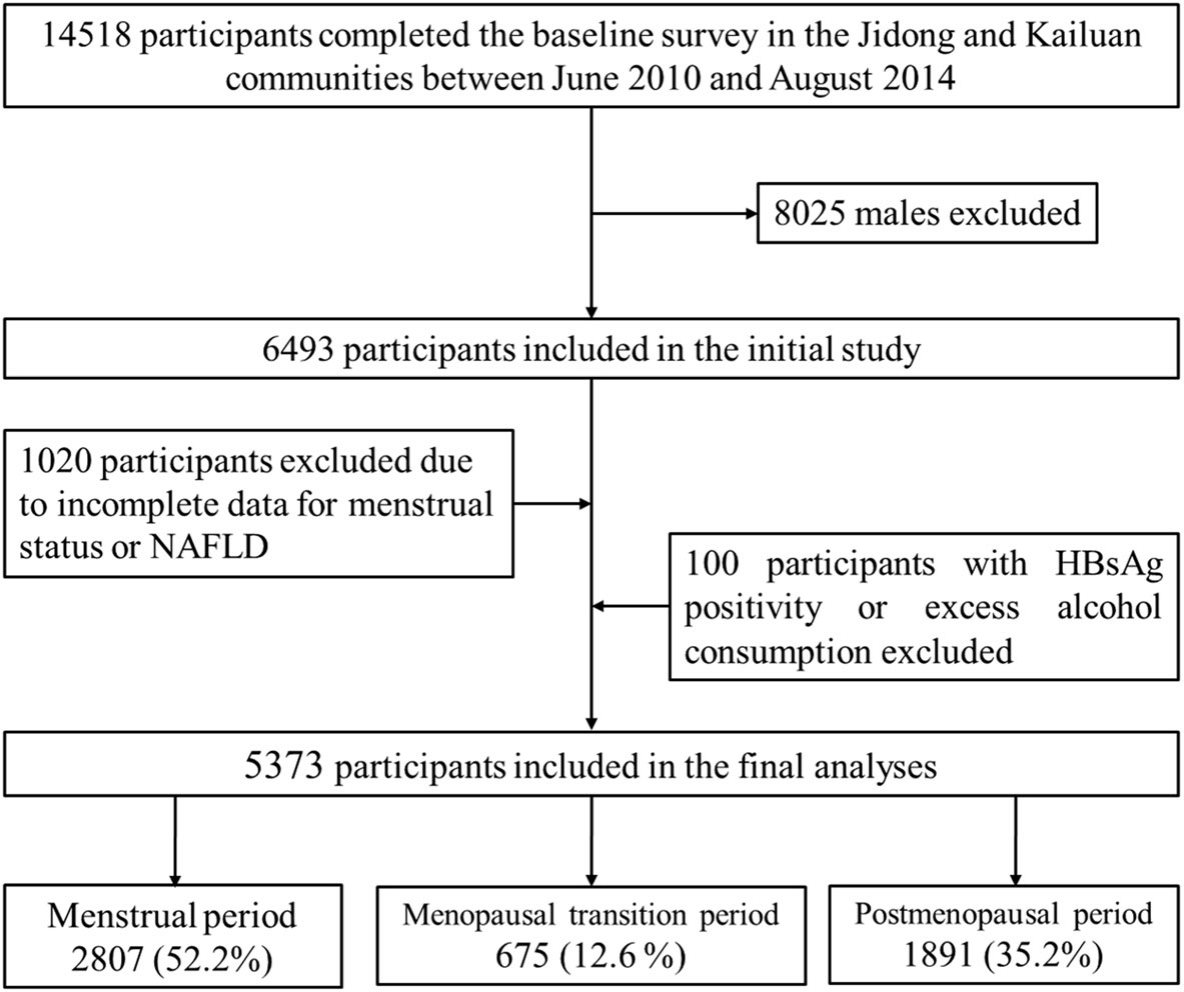
Flowchart of the study. Abbreviation: NAFLD: Nonalcoholic fatty liver disease

The participants in this study were recruited from the Kailuan and Jidong communities in Tangshan City (Hebei Province, northern China) from June 2010 to August 2014. The geographic locations of Kailuan and Jidong are shown in Figure S1. Kailuan General Hospital conducted a health examination on the employees (including those who were retired) of the Kailuan (Group) Co. Ltd. (a large coal mining industry) and their dependents, and finally, 5440 people agreed to the study and completed the questionnaire. Jidong Oilfield Hospital provided the same health examination to the employees (including retirees) of Jidong Oilfield Inc. (a large oilfield) and their families, and 9078 participants agreed to the study and completed the questionnaire. A total of 14,518 people participated in the cohort study. After a detailed evaluation according to the inclusion and exclusion criteria, 5373 women were included in this study, 1360 of whom underwent pericardial fat volume (PFV) testing. The exclusion criteria for this study population were as follows: (I) male sex; (II) a lack of reported menstruation information; (III) incomplete information regarding nonalcoholic fatty liver disease (NAFLD); (IV) hepatitis B surface antigen (HBsAg) positivity; and (V) excessive alcohol consumption (Fig. 1).

### Assessment of obesity

All participants underwent height, weight, waist circumference (WC) and hip circumference (HC) measurement and abdominal ultrasonography by an experienced physician who was blinded to the clinical presentation and laboratory findings. The body mass index (BMI) was calculated as the weight (in kg) divided by the square of the height (in m^2^) and was used to define general obesity (BMI ≥28 kg/m^2^). The waist-to-hip ratio (WHR) was calculated as the WC divided by the HC and was used to assess central obesity (defined as a WHR of > 0.85 for women) according to the criteria proposed by the World Health Organization (WHO) [13]. According to information from the Chinese Association for the Study of Liver Disease and the Asia-Pacific Working Party on NAFLD, diagnosis of NAFLD was based on the presence of at least two of the following three abnormal findings [14, 15]: diffusely increased echogenicity of the liver relative to the kidney, poor visualization of intrahepatic structures, or ultrasound beam attenuation after ruling out excessive alcohol consumption and other liver diseases. Abdominal ultrasonography was performed with a high-resolution B-mode topographic ultrasound system with a 3.5 MHz probe (ACUSON X300, Siemens, Germany) by radiologists who were blinded to the laboratory examination findings. A total of 1360 participants underwent noncontrast chest computed tomography (NCCT) for the quantification of PFV [16]. Visceral obesity was defined as the presence of NAFLD and increased PFV.

### Assessment of menstrual status

Menstrual status was classified into the menstrual period, menopause transition period and postmenopausal period. The 3 periods were defined as follows [17]: the menstrual period was defined as the presence of regular menstrual cycles with ≤7 days of menstruation within a span of 22 to 35 days; the menopause transition period was defined as a change in the length of the menstrual cycle such that menstruation occurred for ≥7 days for at least two consecutive menstrual cycles or amenorrhea was experienced for 3 to 11 months; and the postmenopausal period was defined as the presence of spontaneous or surgical menopause for more than 1 year. The information on menstruation status was collected based on the participants’ self-reports.

### Covariates

Questionnaires were used to collect demographic information, history of disease, lifestyle information, and drug history. Clinical characteristics and biochemical indicators were assessed at Jidong and Kailuan General Hospital. The covariates included age, sex, education level, per capita monthly income, marital status, smoking status, alcohol consumption, physical activity level, age at menarche, oestrogen replacement therapy status, parity, blood pressure, fasting blood glucose (FBG), total cholesterol (TC), high-density lipoprotein cholesterol (HDL-C), low-density lipoprotein cholesterol (LDL-C), and triglycerides (TG). Methods for assessing covariates have been described in a previous study [18]. Hypertension was diagnosed as a diastolic blood pressure ≥ 90 mmHg or systolic blood pressure ≥ 140 mmHg, use of antihypertensive drugs or a self-reported history. Dyslipidaemia was defined as current treatment with lipid-lowering drugs, a history of dyslipidaemia, or serum levels of TC ≥ 5.18 mmol/L, TG ≥ 1.7 mmol/L, HDL-C < 1.04 mmol/L, or LDL-C ≥ 3.37 mmol/L. Diabetes mellitus was defined as current use of insulin or oral hypoglycaemic therapy, a self-reported history, or FBG ≥ 7.0 mmol/L (126 mg/dL).

### Implementation

Before conducting this study, we recruited investigators and physicians in advance. In order to ensure objectivity and authenticity, all investigators and physical examination doctors received strict and standardized professional training before the implementation of the project. An investigator or doctor was assigned to each survey item, and all survey subjects were selected strictly in accordance with the random sampling method.

### Statistical analyses

All continuous variables in our study were normally distributed. Continuous variables are presented as the mean ± standard deviation (SD) and were compared using one-way ANOVA. Categorical variables are described as frequencies and percentages and were evaluated with chi-square tests. Logistic regression was used to analyze the associations between general obesity, central obesity or NAFLD and menstrual status with odds ratios (ORs) and 95% confidence intervals (CIs). The association between PFV and menstrual status was determined by linear regression with an unstandardized β-coefficient and the standard error (SE). The adjusted variables included age, education level, per capita monthly income, marital status, smoking status, alcohol consumption, physical activity level, age at menarche, oestrogen replacement therapy status, parity, hypertension status, diabetes, dyslipidaemia, and TC, TG, HDL-C and LDL-C levels.

All statistical tests were 2-sided, and statistical significance was defined as *P* < 0.05. SAS software, version 9.4 (SAS Institute Inc., Cary, NC, USA) was used for statistical analyses.

## Results

### Participant characteristics

Of the 5373 participants in the study, the proportions of women in the menstrual, menopausal transition, and postmenopausal periods were 52.2, 12.6 and 35.2%, respectively. The characteristics of the participants are summarized in Table 1. Age, education level, per capita monthly income, marital status, physical activity level, smoking status, age at menarche, oestrogen replacement therapy status and parity significantly differed among women in the different menstrual status groups (*P* < 0.001). The prevalence rates of hypertension, diabetes and dyslipidaemia were higher in postmenopausal women than in women in the other two groups (*P* < 0.001). Women in the postmenopausal and menopausal transition groups had a higher BMI, WC, HC, WHR, and PFV, along with higher levels of TC, TG and LDL-C than premenopausal women (*P* < 0.001).

**Table 1:** Basic characteristics of the participants stratified by menstrual status

Characteristics	Total	Menstrual period	Menopausal transition period	Postmenopausal	*P*
Number, n (%)	5373	2807 (52.2)	675 (12.6)	1891 (35.2)	
Age, years	44.7 ± 12.9	34.5 ± 7.8	44.4 ± 5.5	58.5 ± 7.4	< 0.001
Education level, n (%)					< 0.001
Illiteracy/primary,	292 (5.4)	7 (0.3)	9 (1.3)	276 (14.6)	
Middle school	2438 (45.4)	723 (25.8)	350 (51.9)	1365 (72.2)	
College or above	2643 (56.7)	2077 (74.0)	216 (46.8)	250 (13.2)	
Income, ¥/month, n (%)					< 0.001
**≤** ¥3000	2843 (52.9)	967 (34.5)	375 (55.6)	1501 (79.4)	
¥3001–5000	2208 (41.1)	1611 (57.4)	249 (36.9)	348 (18.4)	
> ¥5000	322 (6.0)	229 (8.2)	51 (7.6)	42 (2.2)	
Marital status, n (%)					< 0.001
Unmarried	241 (5.7)	232 (8.3)	2 (0.3)	7 (0.4)	
Married	4994 (93.0)	2533 (90.2)	656 (97.2)	1805 (95.5)	
Other	138 (2.6)	42 (1.5)	17 (2.5)	79 (4.2)	
Current smoking, n (%)	106 (12.0)	38 (1.4)	20 (3.0)	48 (2.5)	0.002
Current drinking, n (%)	204 (3.8)	139 (5.0)	28 (4.2)	37 (2.0)	< 0.001
Physical activity, n (%)					< 0.001
Inactive	1923 (35.8)	1101 (39.2)	228 (33.8)	594 (31.4)	
Moderately active	957 (17.8)	519 (18.5)	160 (23.7)	278 (14.7)	
Active	2493 (46.4)	1187 (42.3)	287 (42.5)	1019 (53.9)	
Menarche age, years	14.6 ± 1.8	13.9 ± 1.5	14.5 ± 1.6	15.6 ± 1.9	< 0.001
Parity					< 0.001
0	543 (10.1)	518 (18.5)	19 (2.8)	6 (0.3)	
1	3847 (71.6)	2182 (77.7)	618 (91.6)	1047 (55.4)	
≥2	983 (18.3)	107 (3.8)	38 (5.6)	838 (44.3)	
Estrogen replacement therapy, n (%)	134 (2.5)	1 (0.1)	8 (1.2)	125 (6.6)	< 0.001
Hypertension, n (%)	1311 (24.4)	264 (9.4)	136 (20.2)	911 (48.2)	< 0.001
Diabetes, n (%)	317 (5.9)	48 (1.7)	21 (3.1)	248 (13.1)	< 0.001
Dyslipidemia, n (%)	1658 (30.9)	458 (16.3)	201 (29.8)	999 (52.8)	< 0.001
BMI (kg/m^2^)	23.7 ± 3.5	22.8 ± 3.4	23.9 ± 3.4	24.9 ± 3.4	< 0.001
Waist circumference (cm)	81.7 ± 10.0	78.9 ± 9.5	81.3 ± 9.4	86.1 ± 9.3	< 0.001
Hip circumference (cm)	96.3 ± 7.9	94.9 ± 7.7	96.4 ± 7.6	98.3 ± 7.9	< 0.001
Waist-hip ratio	0.8 ± 0.1	0.8 ± 0.1	0.8 ± 0.1	0.9 ± 0.1	< 0.001
General obesity, n (%)	607 (11.3)	205 (7.3)	78 (11.6)	324 (17.1)	< 0.001
Central obesity, n (%)	2577 (48.0)	1052 (37.5)	288 (42.7)	1237 (65.4)	< 0.001
NAFLD, n (%)	1674 (31.9)	566 (20.7)	196 (29.7)	912 (49.1)	< 0.001
PFV (cm^3^)	132.8 ± 100.7	92.5 ± 41.4	95.8 ± 37.7	153.6 ± 115.9	< 0.001
TC (mmol/L)	4.6 ± 1.0	4.2 ± 0.8	4.6 ± 0.8	5.2 ± 1.0	< 0.001
TG (mmol/L)	1.4 ± 1.1	1.2 ± 0.9	1.3 ± 1.1	1.7 ± 1.3	< 0.001
HDL (mmol/L)	1.4 ± 0.4	1.3 ± 0.3	1.5 ± 0.4	1.4 ± 0.4	< 0.001
LDL (mmol/L)	2.5 ± 0.7	2.2 ± 0.5	2.4 ± 0.6	2.8 ± 0.7	< 0.001

Abbreviations: *BMI* body mass index, *NAFLD* nonalcoholic fatty liver disease, *PFV* pericardial fat volume, *TC* total cholesterol, *TG* triglycerides, *HDL-C* high-density lipoprotein cholesterol, *LDL-C* low-density lipoprotein cholesterol

**Fig. 2:**
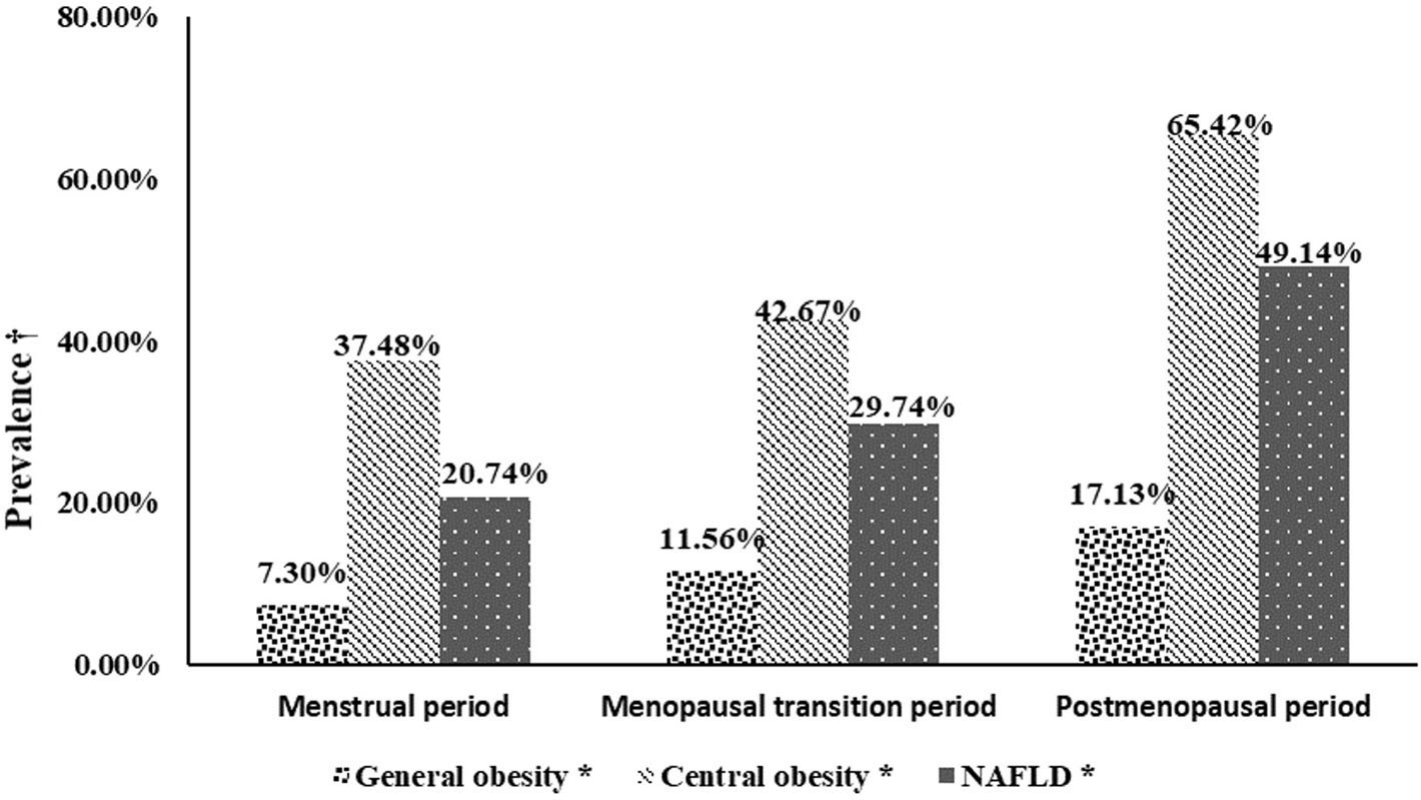
Prevalence of general obesity, central obesity and NAFLD in women with different menstrual statuses. †: prevalence of general obesity, central obesity or NAFLD. ^*^ denotes a significance level of *P* < 0.05. NAFLD = nonalcoholic fatty liver disease

The numbers of participants with general obesity and central obesity were 607 and 2577, respectively. The prevalence of general obesity, central obesity and NAFLD were higher in the postmenopausal group than in the other groups (17.13, 65.42 and 49.14%, respectively) (all *P* < 0.001) (Fig. 2).

### Association of menopausal status with obesity

**Fig. 3:**
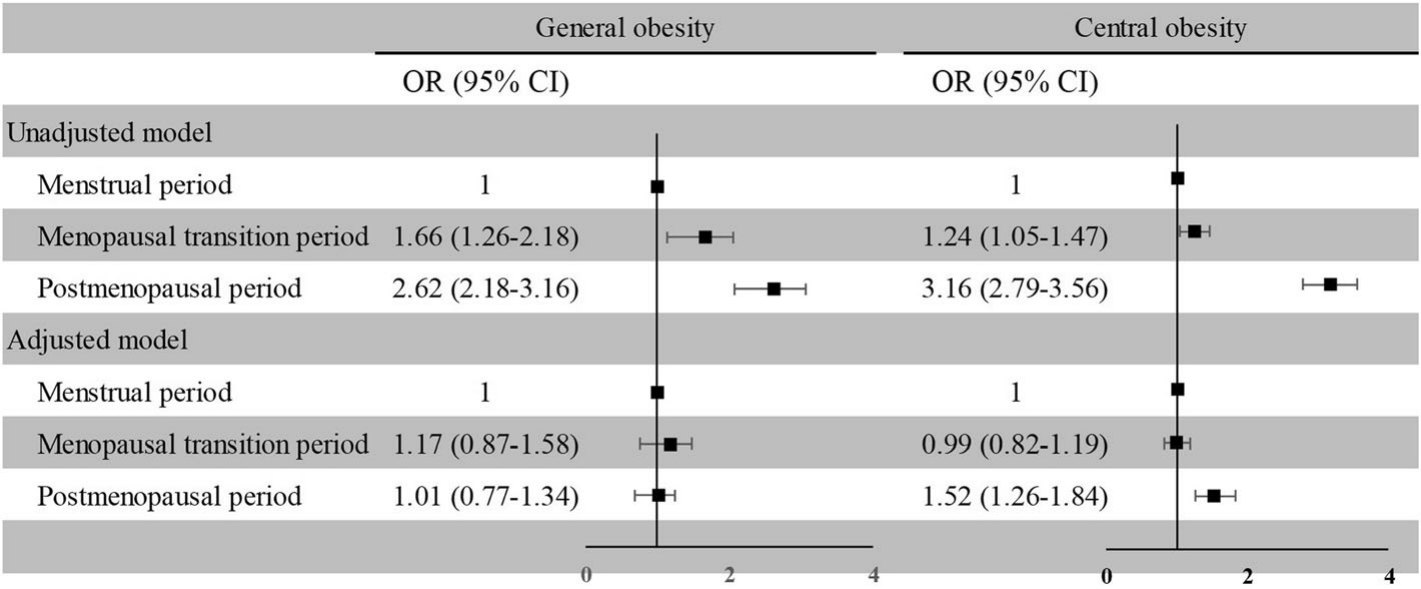
Associations of menstrual status with general obesity and central obesity. The adjusted model included the following covariates: age, education level, income, alcohol consumption, smoking status, physical activity level, marital status, hypertension, diabetes, and TC, TG, LD-C and HDL-C levels

Figure 3 shows the association of menopausal status with general obesity and central obesity. The OR for general obesity in postmenopausal women was 2.62 (95% CI: 2.18–3.16) in the unadjusted model and 1.01 (95% CI, 0.77–1.34) in the fully adjusted model. In the unadjusted model, for women in the menopausal transition period and postmenopausal period, the ORs for central obesity were 1.24 (95% CI, 1.05–1.47) and 3.16 (95% CI, 2.79–3.56), respectively. In the adjusted model, the ORs were 0.99 (0.82–1.19) and 1.52 (1.26–1.84), respectively.

**Fig. 4:**
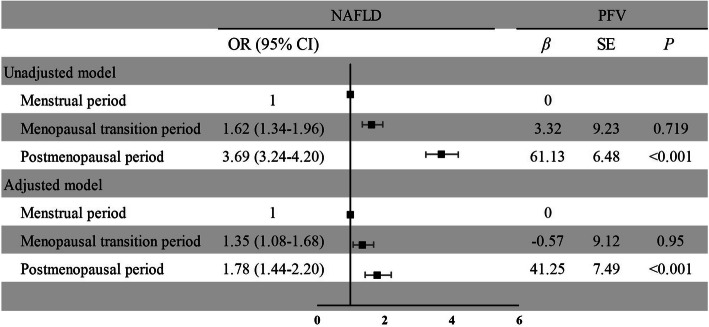
Associations of menstrual status with NAFLD and PFV. NAFLD = non-alcoholic fatty liver disease; PFV = pericardial fat volume. The adjusted model contained the following covariates: age, education level, income, alcohol consumption, smoking status, physical activity level, marital status, hypertension, diabetes, and TC, TG, LD-C and HDL-C levels

The association between menstrual status and visceral obesity is shown in Fig. 4. After fully adjusting for the potential covariates, women in the menopausal transition period and postmenopausal period had higher risks of NAFLD (OR = 1.35, 95% CI: 1.08–1.68; OR = 1.78, 95% CI: 1.44–2.20, respectively). Menopause was a risk factor for increased PFV (β = 41.25, SE: 7.49), but a similar association was not observed for the menopausal transition period (*P* > 0.05).

## Discussion

The findings of this community-based study showed that menstruation status was associated with central obesity and visceral obesity. The risk of central obesity was increased approximately 50% in postmenopausal women compared to women in the menstrual period. Postmenopausal women had an increased risk of visceral obesity. However, menstrual status was not associated with general obesity, although the prevalence of general obesity progressively increased with progressive menstrual status.

One previous study indicated that as women’s menstrual status changes, their weight may increase or decrease, but no association was found between menstruation and the distribution of abdominal fat [19]. However, our findings contradict this research and suggest a positive association between changes in menstrual status and central obesity. A possible reason for this finding is the large sample size in the current study, which might increase the reliability of the results. Similarly, Tremollieres et al. and Ley et al. reported that the amount of abdominal fat increases after menopause and that dual-energy X-ray absorptiometry (DEXA) scans showed that postmenopausal women exhibit more abdominal fat than premenopausal women [20, 21].

We observed that the risk of visceral obesity was significantly higher in postmenopausal women than in women in the menopausal transition and menstrual periods. Lovejoy et al. showed that all women gained fat over time, but only postmenopausal women had a significant increase in visceral fat [22]. Our findings were consistent with the conclusion of that study. In addition, the effect of sex hormones on the distribution of visceral fat in women was consistent with the findings of other epidemiological reports [23].

Several previous studies have assessed the relationship between menstrual status and general obesity [17, 24]. Kim et al. showed a higher prevalence of general obesity in postmenopausal women than in premenopausal women and demonstrated that the serum level of osteocalcin is associated with insulin resistance in postmenopausal women but not in premenopausal women [24]. A longitudinal study also demonstrated a significant association between general obesity and hormone changes in women with different menstruation statuses independent of age, race and smoking status, in contrast to our study results [17]. The participants in that longitudinal study were from the Penn Ovarian Aging cohort, which included a small number of participants, while the participants in the present study were from the Jidong community, and the sample size was larger. Therefore, the differences in the results of these two studies may be due to inconsistencies in the study populations.

The results of previous studies showed that only central obesity and visceral obesity were associated with impaired carbohydrate metabolism, as shown by the higher insulin and glucose levels in obese women than in nonobese women [25, 26]. The evidence regarding the underlying physiological and cellular processes includes the regulation of energy balance and body fat by oestrogen-dependent processes [27]. Oestrogen deficiency and increased androgen activity lead to an imbalance in glucose homeostasis and insulin resistance in postmenopausal women [28, 29]. After menopause, the reduction in the oestrogen level increases the total adiposity and reduces the lean body mass [30]. Therefore, postmenopausal women with low sex hormone levels have an increased likelihood of accumulating body fat [31].

The strengths of our study include the multidimensional evaluation in a community-based cross-sectional study, the large sample size and the investigation of different obesity phenotypes categorized by various sites of fat accumulation. However, some limitations of our study should be noted. First, this cross-sectional study could not identify a causal relationship between menstrual status and obesity. Second, the participants were from the Jidong and Kailuan communities, and residents of these communities have a relatively higher income level than the general Chinese population. The income of these residents has an important influence on their lifestyle, and we did not investigate and analyze calorie consumption and type of diet, which may have increased data deviations and influenced the accuracy of the results. Finally, the Jidong and Kailuan communities are located in a coastal area, and the specific environment may influence obesity and menstrual status.

## Conclusions

The present study demonstrated that menopause is associated with central and visceral obesity and indicated that fat in postmenopausal women is more likely to be deposited in the abdomen and around internal organs than distributed throughout the body. Our study suggests that postmenopausal women should be more conscious of central and visceral obesity and improve their overall health after menopause.

## Supplementary information

**Additional file 1: Figure S1.** The geographic locations of Kailuan and Jidong.

## Data Availability

The datasets used or analysed during the current study are available from the corresponding author upon reasonable request.
