# Supplementary figures and images for: Relationships between menstrual status and obesity phenotypes in women: a cross-sectional study in northern China
Source: BMC Endocr Disord. 2020 Jun 22;20:91. doi: 10.1186/s12902-020-00577-6 (PMC7310131; doi:10.1186/s12902-020-00577-6)

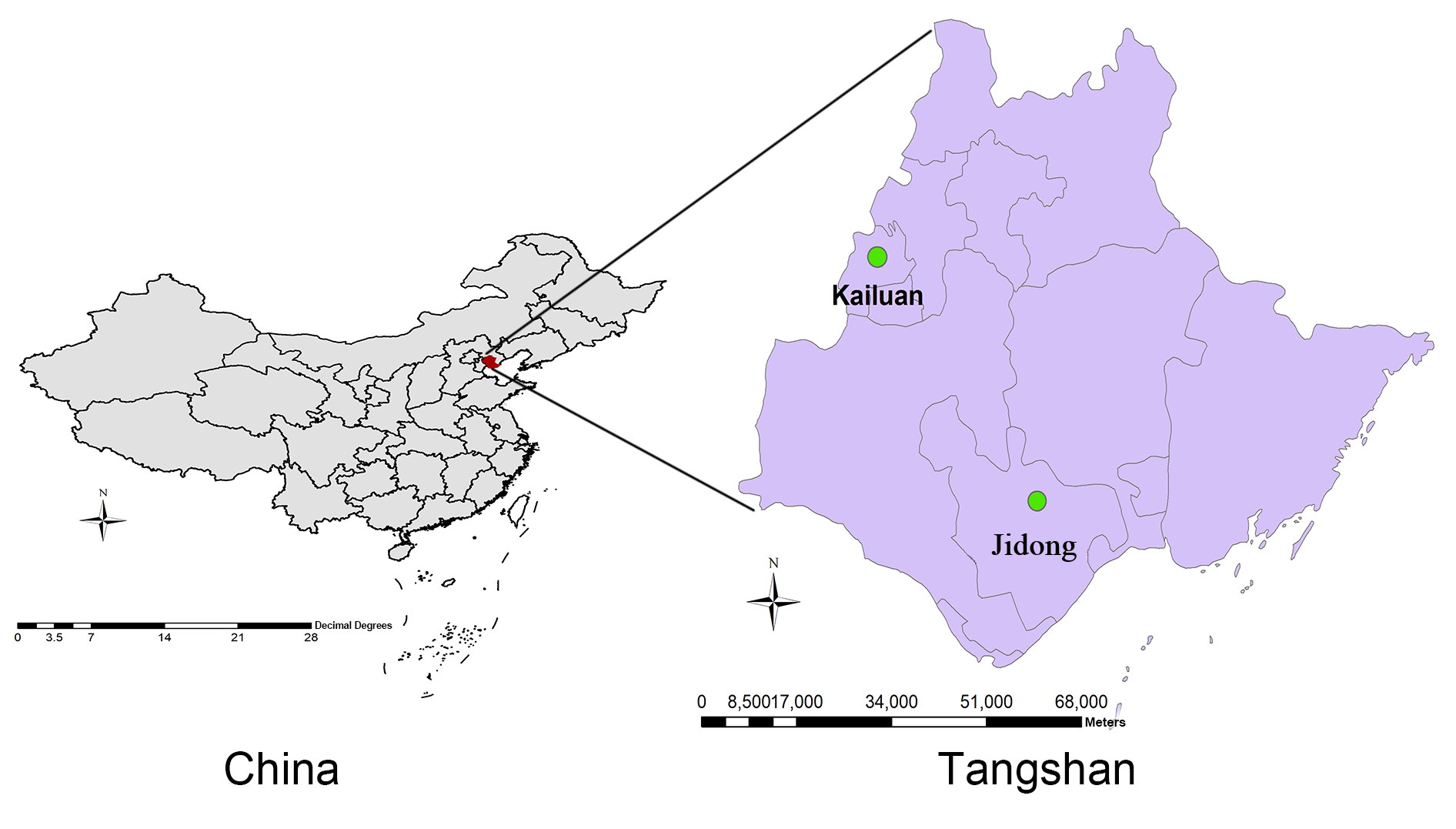

Supplement: Supplementary file 1 — Additional file 1: Figure S1. The geographic locations of Kailuan and Jidong. [file 12902_2020_577_MOESM1_ESM.tif]
